# Common data models and data standards for tabular health data: a systematic review

**DOI:** 10.1186/s12911-025-03267-2

**Published:** 2025-11-13

**Authors:** Melissa Finster, Markus Wenzel, Elham Taghizadeh

**Affiliations:** 1 Department, Fraunhofer Institute for Digital Medicine MEVIS, Max-Von-Laue-Str. 2, 28359 Bremen, Germany; 2 Department, Constructor University, Campus Ring 1, 28359 Bremen, Germany

**Keywords:** Common data model, FAIR-Principles, Data standard, Interoperability, Data harmonization

## Abstract

**Background:**

The use of health data supports knowledge-based decision-making in healthcare. Common Data Models (CDMs) and data standards facilitate the integration of diverse data sources and enable federated analysis by harmonizing data formats and terminologies.

**Methods:**

To determine the best approaches to harmonizing patient data, we undertook a comprehensive literature search, which allowed us to identify the most popular and established CDMs (i2b2, Sentinel CDM, PCORnet CDM, OMOP CDM) and data standards (CDA, HL7 version 2, FHIR, openEHR). We established a set of criteria across the categories of Suitability, Popularity, Adaptability, Interoperability, and Support.

**Results:**

The CDMs and data standards are evaluated based on the defined criteria. Overall criteria the OMOP CDM and FHIR scored best. We highlight the strongest CDM and data standard for each criteria category.

**Conclusion:**

Given the unique characteristics, strengths, and weaknesses of each CDM and data standard, no single global representation can be selected. To promote broad adoption of CDMs and data standards, it is essential to enable transformation between different representations and utilize various formats within a single tool to facilitate their interoperability. Only then seamless data exchange and research across borders can be achieved.

**Clinical trial number:**

Not applicable.

**Supplementary information:**

The online version contains supplementary material available at 10.1186/s12911-025-03267-2.

## Background

**Fig. 1:**
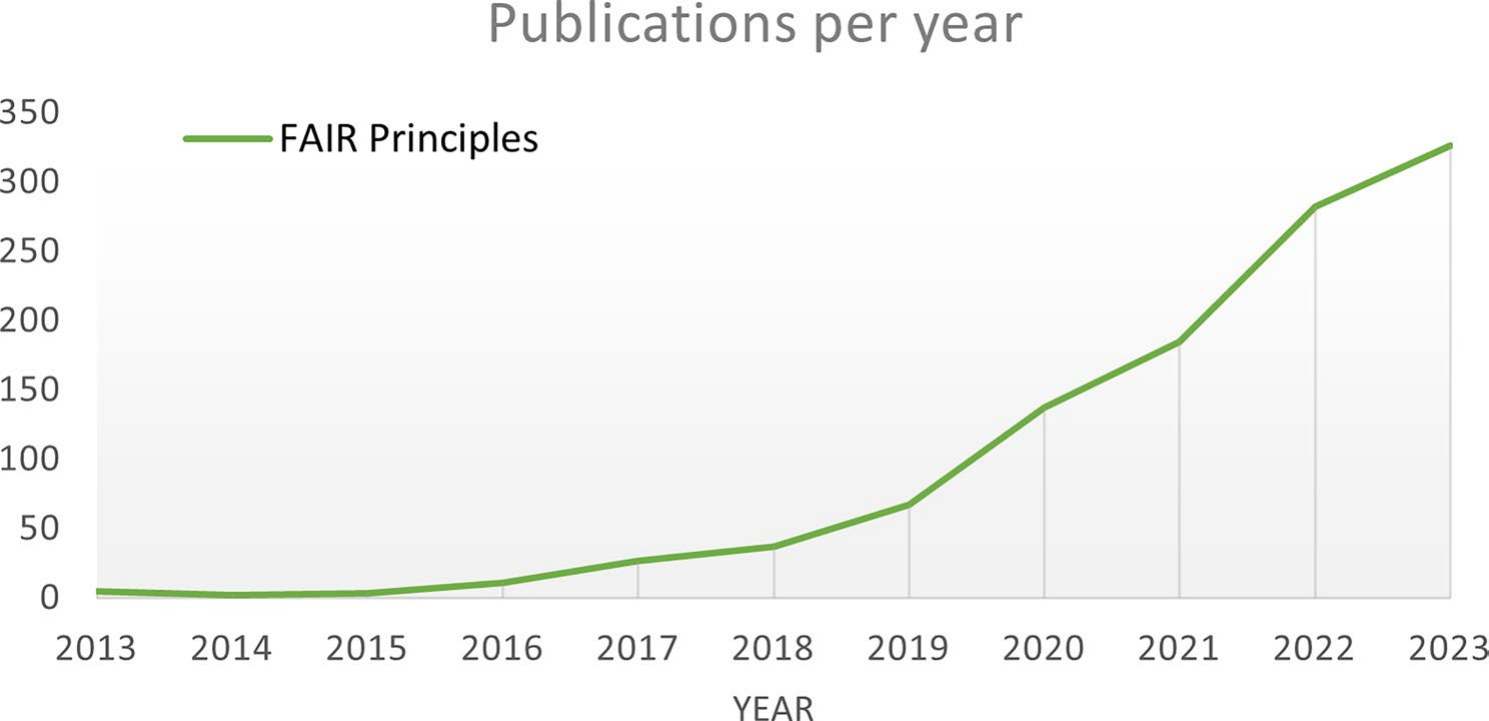
A keyword search of “fair principles” on scopus [7]. The number of publications focusing on fair principles increased strongly within the last years

The use of health data has the potential to significantly improve patient-centered care [1]. This is particularly important given the many challenges faced by healthcare providers and researchers in exchanging health data and conducting large-scale studies across multiple institutions [2]. As such, it is becoming increasingly clear that research on medical data beyond institutional borders is crucial for identifying risk groups, establishing a decision-making framework for pandemic situations, and detecting adverse drug reactions, among other aspects [1, 3]. Moreover, the necessity for a Common Data Model (CDM) for federated learning is emphasized [4]. To achieve these goals, it is necessary to facilitate the reuse and sharing of health data across borders, research institutions and different data providers, including hospitals and medical practices. However, the realization of this goal is not without its difficulties. Various formats, terminologies, and information scopes of collected data make the process of data sharing and reuse highly complex [5]. Moreover, the need to safeguard patient data privacy and security complicates the process. These challenges are being addressed through the development and implementation of FAIR (findability, accessibility, interoperability, and reusability) principles [6]. To this end, there has been a growing interest in the topic of FAIR principles, as evidenced by the increasing number of publications on the subject; see Fig. 1. By highlighting the strengths and weaknesses of various CDM and data standards, we take a step towards the FAIR principles.

A data model represents data elements, its properties and relations, and it serves as a blueprint for organizing data in a structured way. It includes a unified set of metadata that harmonizes data from various sources in a standardized manner. They enable consistent information content across different applications and organizations, promoting integrated and coordinated data, as well as facilitating the federation of research analyses and the aggregation of results. If a data model is a conceptual framework that goes beyond a single use case, it is referred to as a CDM. [8–10].

Data standards can be categorized into two main types: syntactic standards and semantic standards. Semantic standards focus on the meanings of terms, such as specific terminologies. For example, the Systematized Nomenclature of Medicine Clinical Terms (SNOMED CT) is a widely recognized and commonly used semantic standard. CDMs often incorporate these semantic standards as standard concepts, significantly enhancing the interoperability of diverse data sources.

Data standards facilitate information flow within national health information infrastructures, enabling clinical and patient safety systems to effectively collect, share, retrieving, and integrate data. A data standard must address the following: defining data elements, standard formats for electronically encoding these data elements, medical terminologies for classification and coding, and methods for knowledge representation for decision support [11]. In this paper the focus lies on the syntactic standard, which defines structure and format, though possible semantic inclusions are discussed for both, syntactic standards and CDMs. By using CDMs and data standards, interoperability can be achieved and data can be shared among different systems [8, 12–20].

Instead of trying to separate the treatment of CDMs and Data Standards, we acknowledge their tight interdependencies and present them with their complementary roles. We advocate for the necessity of bridging these two domains, as their joint application is crucial for effective data management. While the criteria proposed by [15, 21–23] are invaluable, we elaborated popularity, data exchange, and extract, transform and load (ETL) processes more detailed. Transitioning from one data standard or CDM to another can be costly in terms of both time and resources, and may also lead to potential information loss. Therefore, selecting a widely adopted data standard or CDM that allows for easy transformation is of paramount importance. Additionally, we have taken a holistic approach by not relying on a single data set or source type to evaluate the CDMs and data standards. Instead, we conducted a thorough literature search and systematically reviewed the findings. This methodology allows us to encompass a diverse array of use cases, data sources, health systems, and expert fields. Furthermore, we acknowledge that invaluable findings represented in [15, 21–23] may have been outdated, as the field has evolved with new models, tools, and networks.

On these grounds, we discuss CDMs and data standards identified by a literature search. In Section “Methods” the identified CDMs and data standards, are briefly introduced. We separate them based on their purpose into two groups: CDMs for storing data and data analysis (Section “CDMs for Storing Data and Data Analysis”), and data standards for data acquisition and data exchange (Section “Data Standards for Data Acquisition and Data Exchange”). After an initial overview of their characteristics, we present in Section “Criteria Catalog to assess the CDMs and Data Standards” a set of criteria organized into five categories: Suitability, Popularity, Adaptability, Interoperability, and Support. We evaluate each CDM and data standard against these criteria and present the results in Section “Results”. In Section “Discussion” we discuss and illuminate the derived Results.

## Methods

We conducted a comprehensive literature search to identify the most popular CDMs and data standards based on their prevalence in Scopus [7]. Following, we briefly introduce the CDMs in Section “CDMs for Storing Data and Data Analysis” and data standards in Section “Data Standards for Data Acquisition and Data Exchange” [15].

### Assessment approach

**Fig. 2:**
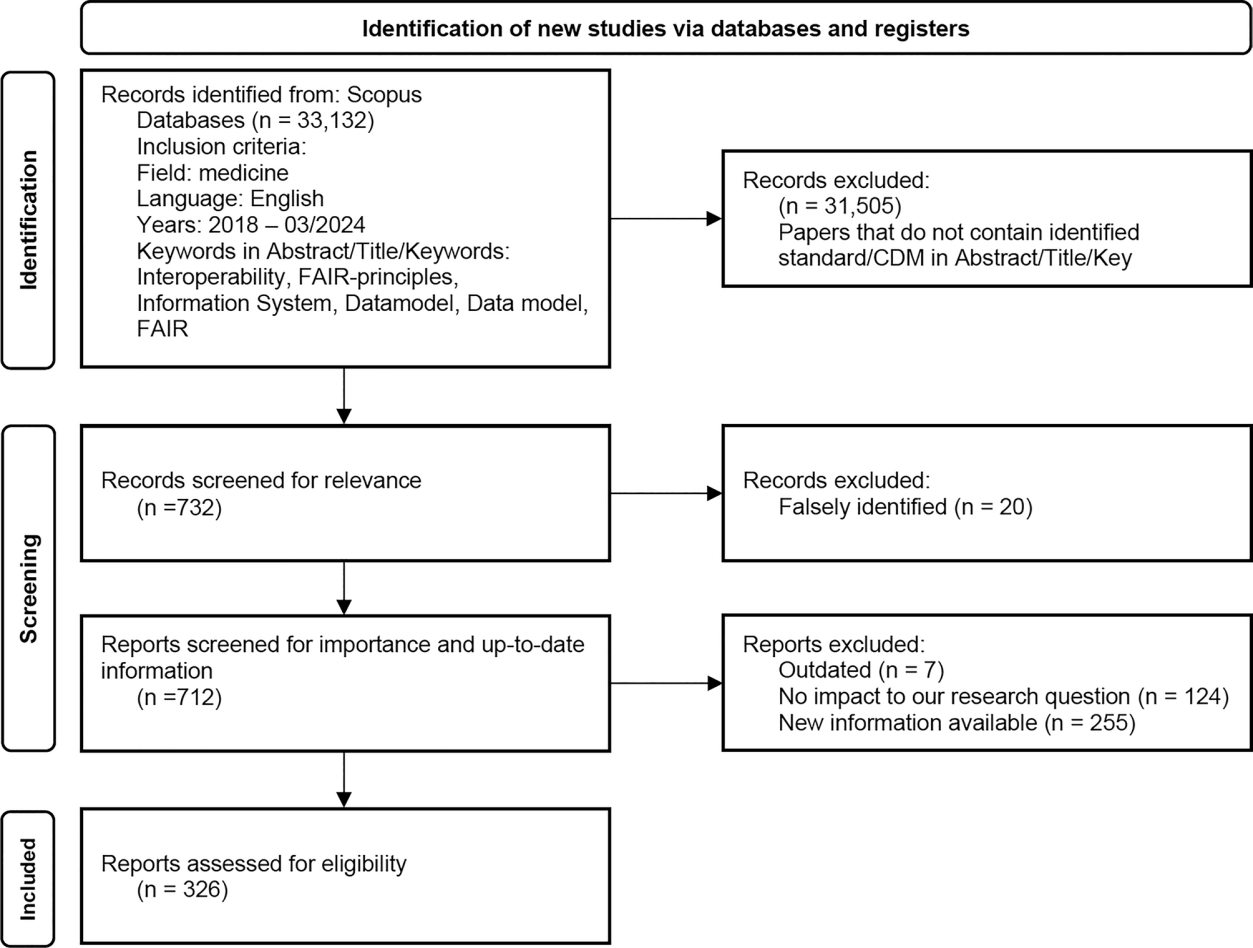
Approach of selecting collection of literature

We undertook a comprehensive review of relevant literature, with detailed information provided in the Appendix A. The identified papers were filtered and exclusion criteria were applied; see Fig 2. For each CDM and data standard, a list of references was collected. If a paper covers multiple data standards or CDMs, it was incorporated into the relevant CDMs and data standards.

The CDMs and data standards have been randomly assigned among the authors, allowing us to review our designated list of literature. We have documented the information and references that met the established criteria. The final assessment of the literature was conducted by the authors, based on the collected data.

### CDMs for storing data and data analysis

We first introduce the CDMs for storing and analysis, namely the Sentinel CDM (SCDM), PCORnet CDM, i2b2 CDM and the OMOP CDM. Each of these CDMs is a development targeting a specific research question.

#### Sentinel

In 2007, the US Food and Drug Administration (FDA) requested that drugs be tested based on real-patient data after the drug has been released to the market [24–27]. To this end, the FDA launched the Sentinel Initiative and the Mini-Sentinel CDM was developed in 2008 [28, 29]. Over time, it evolved into the full SCDM. The SCDM focuses on rapid adverse drug event detection, drug safety, and monitoring in the pharmaceutical industry and had been used for hundreds of privacy-preserving analyses [30]. The SCDM structures data in tables, organized in domains. The SCDM and its tools are SAS based [31]. The latest extension of the SCDM was released in 2022 and can be accessed from the Sentinel repository [32].

#### Pcornet CDM

The Patient Centered Outcomes Research Network (PCORnet) CDM was developed based on the Mini-Sentinel [33, 34] and is funded by the Patient Centered Outcomes Research Institute [35]. With the PCORnet CDM, first released in 2014, PCORnet aims to conduct studies across multiple networks. Its latest version can be accessed on [36]. The table based model can be queried with SAS or SQL.

#### i2b2

The Informatics for Integrating Biology & the Bedside (i2b2) model is an openly accessible CDM used for data integration and standardization. It was developed in 2004 and has since been used by over 200 institutions [37, 38]. The i2b2 model is constructed as a star schema, i.e., a schema with one central table surrounded by linked tables. Model description, code links and further documentation is available at [39].

#### OMOP CDM

The Observational Medical Outcomes Partnership (OMOP) CDM was established in response to the same request from the US FDA as Sentinel [10, 40]. Yet, it has expanded to include Electronic Health Records (EHR), medical notes, and other types of health-related data. As a result of the OMOP, a public-private partnership, the Observational Health Data Sciences and Informatics (OHDSI) was established in 2014 [40–42]. In 2011, Overhage et al. identified the first requirements of the OMOP CDM [43]. The SQL-based model is divided into domain-oriented concepts.

### Data standards for data acquisition and data exchange

Data standards are used in clinical daily life to record, exchange, and request information on patient level. The four introduced data standards are syntactic standards, i.e., defining the structure and format of the data, and are freely available.

#### HL7 version 2

Health Level Seven (HL7) is a non-profit, American national standards institute [44]. HL7 has developed multiple data standards. The data standard HL7 version 2 supports hospital workflows from patient registration to hospital logistics. HL7 version 2 is used in more than 35 countries and by 95% of the US healthcare organizations [44]. It is the most widely used healthcare interoperability standard and is heavily utilized by hospitals and healthcare IT suppliers [45].

#### CDA

The Clinical Document Architecture (CDA), released in 2000, is a version of the HL7 version 3, Reference Information Model, that was redefined and enhanced [46]. The CDA is an XML-based document markup standard that specifies the structure and semantics of clinical documents [44, 45, 47–49]. The main objective of CDA is standardizing the clinical documents that already exist as free text, and its main purpose is to exchange information among those involved in the care of a patient [50]. A CDA document consists of a header and a body. The header provides structured metadata about the document itself, like the context in which the document was created [50]. The header serves to facilitate clinical document exchange and management within and across institutions, and over the course of a patient lifetime. The body contains the informational (factual) statements that make up the actual content of the document.

#### FHIR

The Fast Healthcare Interoperability Resources (FHIR) were developed by HL7 in 2011. The human-readable standard describes its format and components, also known as resources [45, 51, 52]. FHIR serves in the daily clinical routine by easing the exchange of patient data between parties [19, 53, 54]. With a Representational State Transfer (RESTful) Application Programming Interface (API) the system can be fed with health information. Information can be functional operations (services), a fixed set of information (message), or a fixed package of information (document). As a web API, FHIR has a strong foundation in web standards, such as XML, JSON, HTTP, and OAuth.

#### openEHR

The openEHR public standard was established in 2003 by the non-profit organization openEHR International and standardized to the EN/ISO 13606 standard series by CEN and ISO. OpenEHR has more than 3200 users worldwide and approximately 30 partners [55]. It is used for reporting EHR as well as research analytics [17, 56]. It applies a 3-level approach, which contains of the reference model, reusable content element definitions, and context-specific data set definitions [57–61]. The content element definitions are building blocks, known as archetypes. Combining archetypes forms a template, which defines a use case specific data structure [62–64]. Examples like the pathology report template can be found on the openEHR website [55]. Its implementation technology specifications include XSDs, JSON-schema, and REST APIs [55].

### Criteria catalog to assess the CDMs and data standards

To evaluate the introduced CDMs and data standards, we first define criteria to compare the CDMs and data standards. We have carefully selected three review papers [15, 22, 23] along with the guidance principles from the European Medicines Agency (EMA) [21] that define criteria for CDMs and data standards. This selection allows us to establish a comprehensive set of criteria to evaluate both CDMs and data standards.

The criteria are detailed in Table 1. The phrasing and grouping of criteria has been considered along the following dimensions. Notably, use case-based criteria have been excluded from our analysis, as our work does not rely on a single use case. Additionally, criterion “costs” has not been included, given that all CDMs and data standards are freely accessible. Yet, the SCDM is based on the fee-based Statistical Analysis System (SAS). While the criterion of scalability was mentioned by González-Ferrer et al. [23], it was not evaluated in their work. The criterion for backend-frontend communication was only addressed by González-Ferrer et al. [23]. All other relevant criteria are thoroughly encompassed in our analysis.

**Table 1:** Overview of selected criteria within a collection of review paper and guidance principles [15, 21–23]

Criteria	Source				
	González-Ferrer	Garza	Schneeweiss	EMA	Our
	et al. [23]	et al. [15]	et al. [22]	[21]	**paper**
Suitability	$\surd$	$\surd$			$\surd$
Spread	$\surd$	$\surd$			$\surd$
Available networks	$\surd$			$\surd$	$\surd$
Data exchange (ETL)	$\surd$				$\surd$
Freedom to extend	$\surd$	$\surd$	$\surd$	$\surd$	$\surd$
Evolution and maintenance		$\surd$			$\surd$
Terminologies and concepts	$\surd$	$\surd$		$\surd$	$\surd$
Governance	$\surd$			$\surd$	$\surd$
Data validation			$\surd$	$\surd$	$\surd$
Ease of Use	$\surd$	$\surd$	$\surd$	$\surd$	$\surd$
Tools	$\surd$		$\surd$	$\surd$	$\surd$
Version Control		$\surd$		$\surd$	$\surd$
Integrity (use case based)		$\surd$			not applicable
Querability (use case based)		$\surd$			not applicable
Cost			$\surd$		not applicable
Scalability	$\surd$ (not measured)				
Backend-frontend	$\surd$				
communication					

After a thorough review of the proposed criteria, we have identified the following criteria organized into five distinct categories:

1. **Suitability to various data sources and use cases**: The chosen CDM or data standard should address a wide range of data sources (e.g., EHR, claims data, survey data) to enable cross-cutting use cases and facilitate data reuse.
2. **Popularity**
  3. **Spread**: Even the most suitable CDMs can only be established if it is widely known and used.
  4. **Data networks and secure data exchange**: Data networks enable cross institutional research, while satisfying data protection and security declarations [65]. Data networks with many data partners influence the choice of the CDM for storing data and data analysis. On the other hand, data standards should enable an easy, fast, and secure exchange of data between parties. Therefore, standards which intend to exchange sensitive data must provide security standards.
  5. **Data exchange with other CDMs and data standards**: The ability to easily transform data between CDMs increases the number of data sources that can be utilized in a study.

6. **Adaptability**
  7. **Freedom to extend**: The ability to extend or adapt the CDM minimizes information loss.
  8. **Evolution and maintenance**: Continuous evolution and regular maintenance are required to accommodate changing healthcare demands and prevent errors.

9. **Interoperability**
  10. **Terminologies and concepts**: Mapping data to standard concepts enables easy unification of data from various sources but may require significant resources and effort, potentially leading to information loss.
  11. **Governance**: Strong model governance eases running the same analysis on various data sources or joining data from several sources. It is in conflict with Criterion 3a.
  12. **Data validation**: Comprehensive validation tools help to keep data quality.

13. **Support**
  14. **Ease of use**: The CDM should be understandable and usable by individuals with different backgrounds, such as data collectors, maintainers, and researchers. Transparent and clear definitions of concepts and rules are necessary to handle data transformation consistently. A strong community support with experience in edge cases and common practices is crucial.
  15. **Tools**: Provided tools to support developer and user, for instance, to generate the CDM or message, to develop an ETL process, or to create analysis queries.
  16. **Version control**: The ability to track changes in patient history. For instance, conducting a study with the cohort based on their residence might be inaccurate without versioning.

## Results

Based on the defined criteria, we evaluate each of the CDMs and data standards listed in Section “CDMs for Storing Data and Data Analysis” and Section “Data Standards for Data Acquisition and Data Exchange”. The results of the CDMs are summarized in Table  2, while the evaluation of the data standards is shown in Table 3. In the subsequent Section “Discussion” we discuss and justify all CDM and data standard ratings we have aggregated in Tables 2 and Table 3. For easier accessibility, we have cross-linked the respective detail discussions from the table entries.

**Table 2:** Overview of CDM meeting defined criteria

Criterion	SCDM	PCORnet	i2b2	OMOP
1 Suitability	+	+	+	++
2a Popularity: Spreading	++	++	++	++
2b Popularity: Data Networks	++	++	++	++
2c Popularity: ETL	0	++	++	++
3a Adaption: Freedom to extend	+	+	++	+
3b Adaption: Evolvement	++	++	++	++
4a Interoperability: Terminologies	+	++	+	++
4b Interoperability: Governance	+	++	+	++
4c Interoperability: Interop.: Data validation	++	++	+	++
5a Support: Ease of use	++	++	+	+
5b Support: Tools	++	+	++	++
5c Support: Version control	0	+	0	+

**Table 3:** Overview of data standards meeting defined criteria

Criterion	HL7 v 2	CDA	FHIR	openEHR
1 Suitability	++	++	++	++
2a Popularity: Spreading	++	++	++	++
2b Popularity: Data Networks	+	+	++	++
2c Popularity: ETL	+	+	++	++
3a Adaption: Freedom to extend	++	++	++	++
3b Adaption: Evolvement	+	++	++	++
4a Interoperability: Terminologies	++	++	++	++
4b Interoperability: Governance	+	+	++	++
4c Interoperability: Data validation	++	++	++	++
5a Support: Ease of use	+	+	++	+
5b Support: Tools	+	++	++	++
5c Support: Version control	0	+	++	++

As shown in Table 2, the OMOP CDM scored the highest in the overall assessment of the CDMs with a score of 21+, followed by PCORnet with 20+, i2b2 with 17+, and SCDM with 16 + . Criterion 3a (Freedom to extend) is led by i2b2, while Criterion 5a is led by both PCORnet and SCDM. Multiple criteria have received identical scores.

Table 3 shows that FHIR achieved the maximum score (24+) in each category for the data standards. OpenEHR is presented as a strong alternative to FHIR with a score of 23 + . As a predecessor of FHIR, CDA scored 19+, while HL7 v2 scored 16+, indicating that they are less competitive in these categories. Several criteria have obtained identical scores.

## Discussion

*Criterion 1 (Suitability to various data sources and use cases)*

**Fig. 3:**
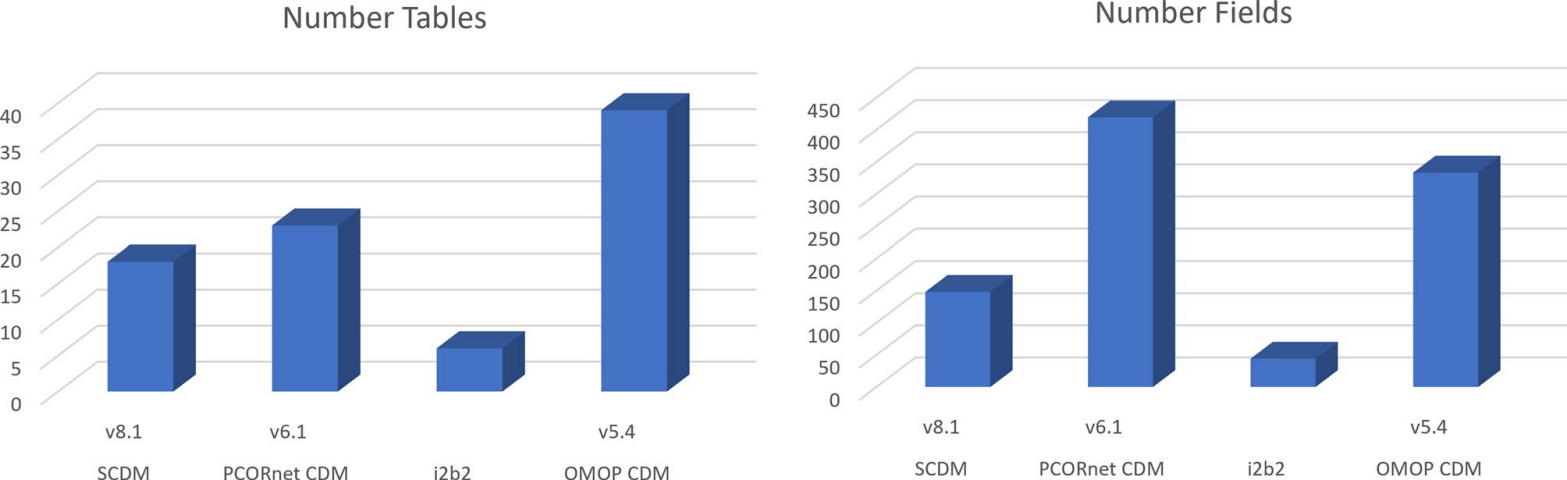
The number of tables (left) and number of fields (right) in the CDMs. The omop CDM counts the most tables and PCORnet CDM the most fields

In Table 4 an overview of possible data sources and reported use cases is given. Additionally, Fig. 3 shows the number of tables and fields of the four considered CDMs. The number of tables and the number of fields are first indicators of the domain scope and details. Yet, it is not a direct measurement. Garza et al. [15] obtained a comparable result. They found that the OMOP CDM achieved the highest domain coverage, followed by the Study Data Tabulation Model, which is not part of our study. PCORnet rated third, while SCDM came in fourth. However, unstructured data cannot be included in the PCORnet [93].

**Table 4:** Overview of CDM suitability. suitability of data types that can be stored and reported use cases

CDM	Data	Common Use Cases
SCDM	EHR, pharmacy service, laboratory results, stroke registries, Medicare Fee-for-Service, text, link to external sources possible, e.g., registries [27, 66–71]	Observational cohort studies, embedded randomized trials, drug safety and monitoring, drug exposure analysis of neonates and infants, analyses and prediction of relevance to nephrology, hospital outcomes (e.g. hospitalized stroke) [30, 68, 69]
PCORnet	EHR, administrative data, inpatient, outpatient, emergency department, ancillary service, patient-reported outcomes, area-level social and behavioral determinants [72, 73]	Computable phenotypes for resistant hypertension, stable controlled hypertension, longitudinal EHR studies, drug safety and monitoring, monitoring rates of COVID-19 illness, complications, and correlations [72, 74]
i2b2	EHR, biospecimen data, case report, survey data, cancer registry, anatomic pathology, clinical findings, oncological medication, unstructured text and reports, images, and genomics [39, 75–77]	Clinical trials, monitoring drug safety, epidemiology research, phenotyping, genotyping, registry studies, anatomic pathology studies, medication studies [39, 75–78]
OMOP	Clinical data, health system data, health economics data, derived elements, molecular markers, hematology data, notes, images [15, 79–81]	Analyzing longitudinal data, identify high-risk patients, treatment pathway planning for pediatric epilepsy, studies on depression, hypertension, diabetes type II, dementia, drug safety studies, automatic trial matching, predicting risk of smoking score, epidemiology studies, preventing cardio-cerebrovascular disease [10, 15, 41, 59, 80, 82–92]

In Table 5 data types for the standards are listed. Use cases are not considered since they are developed for clinical daily routine and data exchange.

**Table 5:** Overview of data standard suitability. suitability of data types that can be stored and reported use cases

Data Standard	Data	Additional Information
HL7 v2	Clinical data, EHR, laboratory results, genomic data [94]	A message is comprised of segments, which contain mandatory and optional fields [45]
CDA	EHR, laboratory reports, and administrative data, tables, lists, and any relevant multimedia data [46, 48–50, 95, 96]	CDA consists of a header, body, and sections. The structure and semantics is defined in templates [46, 48–50]
FHIR	Human and veterinary care, clinical trials, public health, genomic, lab results, vital sign, medication request, allergy intolerance [45, 97, 98, 98–100]	Approx. 145 resources are categorized into five groups: Foundation, base, clinical, financial, and specialized [45]
OpenEHR	Clinical daily routine, public health data, pharmacy, genomics, clinical registries, EHR, administration [55, 61]	Has a clinical decision-making system [55]

Taking into account the findings presented by Garza et al. [15], we evaluate the SCDM using a ranking of $+$. The minimalist i2b2 CDM also receives a $+$ rating due to its limited number of domains. In contrast, while the OMOP CDM is awarded a $++$, the PCORnet CDM cannot accommodate unstructured data, resulting in a $+$ evaluation for PCORnet. All data standards can hold a wide field of data sources to comply their purpose, evaluated by $++$.

### Criterion 2a (Spread)

Given that the selected CDMs and data standards have been identified based on their popularity, Criterion 2a is trivially satisfied. Further details can be found in Appendix A.

### Criterion 2b (Data networks and secure data exchange)

CDMs are categorized into those for analysis and storing, and data standards. According to the European medicines agency’s report [21] the access to the Sentinel network is restricted to the FDA. Additionally, the Sentinel operations center coordinates the network of Sentinel data partners. The Sentinel Distributed Database operates as a distributed network, ensuring that data remains within institutional firewalls. If patient-level data is shared, it is fully anonymized and restricted to essential information. The data is shared using the PopMedNet platform. If needed, text records can be provided, and data can be linked to external registries [71]. For each query, the Sentinel operation center creates an analytic package, which is executed by the data partners. The aggregated data is then returned to the operation center for final analysis [69, 70]. The Sentinel Distributed Database spans 500.1 million unique patient identifiers from 2000 to 2024 and contains 1.3 billion person-years of data with 22.3 billion pharmacy dispensings and 24 billion unique medical encounters [32]. Within the Precise4Q a harmonization framework was developed and used to integrate various heterogeneous stroke-related datasets from institutions throughout Europe [66]. Huang and colleagues adapted the SCDM to the National Health Insurance Research Database in Taiwan [101]. The Oxford Royal College of General Practitioners Clinical Informatics Digital Hub, ORCHID uses the SCDM as well as the OMOP CDM to satisfy the FAIR principles [102].

Like Sentinel, PCORnet is based on the PopMedNet, too. It consists of eleven research networks and one coordination center. The clinical research networks connect among others 337 hospitals, 3,564 primary care practices, and 1,024 community clinics [36, 72, 103–106]. For data security reasons, data is maintained locally by the institutions [107]. Only requests are sent through the distributed research network query portal and only results are returned [108]. Carnahan et. al used billing data, and EHR of three PCORnet networks (Greater Plains Collaborative, OneFlorida, STAR) for Assessing Use of Molecular-Guided Cancer Treatment. Although some limitations were found, several studies suggest that EHR at PCORnet networks were highly complete for their use case [109]. PCORnet offers encrypted, keyed secure hash tokens to match patient records [110].

i2b2 offers a Shared Health Research Information Network, called SHRINE [111]. On that account, i2b2 offers authentication and authorization based on HIPAA guidelines [39, 75, 112]. Some institutions established SHRINE during projects, e.g., Harvard Catalyst, which was decommissioned and augmented by the ACT Network. Additionally, tranSMART was established for sharing, integration, standardization and analysis of diverse data [39, 113, 114]. i2b2 CDM is used by over 200 organizations worldwide [78, 115]. González et.al [76] report from the project InSite, a global clinical research network, with over 130 healthcare providers, Gardner and colleagues [77] introduce a de-identified i2b2 clinical data warehouse with multiple hospitals and more than half a million patients and Castro et. al [75] introduce the Mass General Brigham Biobank Portal with 125,645 patients. Based on tranSMART and i2b2 Johns et. al introduce a data warehousing portal to provide access to a range of data warehouses [116]. The German project Data Integration for Future Medicine makes use of the i2b2 CDM, as well as of tranSMART. Cross-site selection between university hospitals is based on SHRINE [117]. The Accrual to Clinical Trials network of 21 National Clinical and Translational Science Award sites. It deploys a set of i2b2 repositories and complements the PCORnet [111].

To conduct studies involving multiple institutions, with the OMOP CDM the OHDSI network can be utilized. The OHDSI distributed data network comprises over 331 data sources, containing more than 2.1 billion patient records across 34 countries [118]. For network research studies, researcher check the available databases and get in touch with potential collaborators [42]. Aggregated results are shared across the network while patient-level data remains within each institution. The OHDSI GitHub platform provides access to the analysis and aggregated study results of open OHDSI network studies. The Arachne platform automates the network study process [119]. Since 2020, OHDSI collaborates with the European Health Data and Evidence Network (EHDEN) [10, 120, 121].

Data exchange within all HL7 standards are possible. HL7 version 2 allows the exchange of clinical data among systems using messages. HL7 version 2 does not provide security applications or authentication tools. The standard leaves the implementation to the end-user [51]. Whereas FHIR and CDA offer security features, such as security applications and authentication tools [45, 51, 54, 122]. The FHIR API does not impose specific security rules on operations. However, it includes essential building blocks for encryption, TLS (Transport Layer Security), access control, user management, OAuth (Open Authorization) and provenance tracking to support various security approaches [52, 123]. FHIR enables easy exchange of small pieces of information, i.e., resources [124], while HL7 version 2 and CDA usually exchange whole reports and large amounts of data as message or document. The United States, Canada, Argentina, Brazil, Chile, and Colombia are using FHIR for health data exchange [125]. The World Health Organization recommends FHIR as a standard for structuring SMART guidelines [125].

OpenEHR supports individual and population-level queries and enables federated querying across datastores [126]. It has minimal security policies, with the latest API version covering authentication and authorization. For instance, openEHR supports digital signature and access control [55].

Since CDA and HL7 version 2 usually exchange rather whole exports and due to missing security applications and authentication tools for HL7 version 2, we have evaluated them with $+$, while for all other CDMs and data standards the criterion is completely fulfilled.

### Criterion 2c (Data exchange with other CDMs and data standards)

The adoption of CDMs enables efficient data transformation and collaboration. Three primary approaches can be identified in the ETL landscape:

17. **Direct ETL Processes**: These involve the direct transformation and mapping of data from one CDM to another, such as from i2b2 to FHIR or OMOP. Tools developed for these processes aim to simplify the conversion and ensure data completeness.
18. **Query Translation**: This approach allows users to query data across different CDMs without the need for data transformation. It facilitates unified querying and analysis, maintaining data in its original format while enabling cross-model compatibility.
19. **Layered CDM Integration**: Some tools build upon existing CDMs to create new standards or knowledge graphs, integrating various models into a cohesive framework. This method emphasizes interoperability and the ability to leverage multiple data standards simultaneously.

For a comprehensive list of available ETL tools and processes, refer to Table 6.

**Table 6:** Tools supporting interoperability between CDMs

**Direct ETL Processes**			
**Source**	**Target**	**Example Project(s)**	**Publication(s)**
PCORnet	OMOP CDM		[8, 110]
i2b2	FHIR	CAMP FHIR, i2b2	[19, 39, 127–129]
RDF	i2b2	i2b2	[130, 131]
CDA	OMOP		[132]
FHIR	PCORnet, OMOP		[133]
FHIR	OMOP	MIRACUM	[97, 134–137]
openEHR	i2b2		[64]
openEHR	OMOP CDM		[138]
EHR messages	HL7 v2		[139]
**Query Translation**			
**Source**	**Target**	**Example Project(s)**	**Publication(s)**
i2b2, openEHR	openEHR, i2b2		[140]
OMOP	i2b2		[141]
openEHR, FHIR	FHIR, openEHR		[142]
PCORnet, OMOP	i2b2	SHRINE	[115, 143]
**Layered CDM Integration**			
**Base CDM**	**Layered CDM**	**Example Project(s)**	**Publication(s)**
OMOP	FHIR	FHIR-Ontop-OMOP, OMOPonFHIR	[144, 145]
openEHR	FHIR	GECCO	[63, 146, 147]

In addition to the ETL processes and query translation tools between two CDMs, the Common Data Model Harmonization (CDMH) project aims to standardize various CDMs like PCORNet, i2b2, OMOP, and Sentinel by mapping them to the Biomedical Research Integrated Domain Group (BRIDG) model, facilitating easier sharing and interpretation of research data. The CDMH FHIR Implementation Guide (IG) focuses on translating observational data into FHIR format, enabling efficient data publication through RESTful APIs and leveraging FHIR tools for enhanced data extraction from clinical systems [44, 148, 149]. Table 6 exposes a notable scarcity of solutions for CDA, HL7 version 2, and Sentinel. Furthermore, no solutions are offered for the more generalized solutions: query translation and layered CDM Integration. Leading to an evaluation of $+$ for CDA and HL7 version 2, and a neutral evaluation $0$ for the SCDM.

### Criterion 3a (Freedom to extend)

Criteria 1 address the general defined scope of the CDM, whereas Criterion 3a addresses the ability to extend, which might be a local solution but hamper interoperability (Criteria 4).

In the SCDM project specific data elements can be extracted [21]. However, the project specific data elements are outside the Active Risk Identification and Analysis (ARIA) system [71].

PCORnet offers, beside its core tables, supplementary tables for specific use cases, which do not need to be populated in general [34]. Additionally, user might extend their CDM outside of supplementary tables , e.g., Hornik et. al developed four custom data domains because of their importance to the trial [150].

For OMOP CDM, we also found multiple publications that reported the possibility of extending the model for specific applications, e.g., time-based comorbidity [151], clinical next-generation sequencing data [152], chemotherapy regimens [153], and microbiology lab results [154]. Yet, these extensions can be only used locally. PCORnet as well as OMOP are suggesting to extend the CDM only if it is crucial [34, 42].

The i2b2 CDM is more flexible than the other three CDMs [19, 76, 151]. Making adaptations is easy and intended [75, 78, 115].

CDA, FHIR and openEHR are intended to be adapted [45]. HL7 version 2 offers the z-Segment, which can be designed by the user to satisfy user-specific needs [45]. FHIR is even more flexible than its predecessors [53, 125].

Garza et al. [15] classify the extensibility of new domains within SCDM, PCORnet, and OMOP as straightforward. However, because these extensions might restrict the use of other tools and networks, we evaluate them with $+$. i2b2 and the four data standards are rated with $++$.

### Criterion 3b (Evolvement and maintenance)

**Fig. 4:**
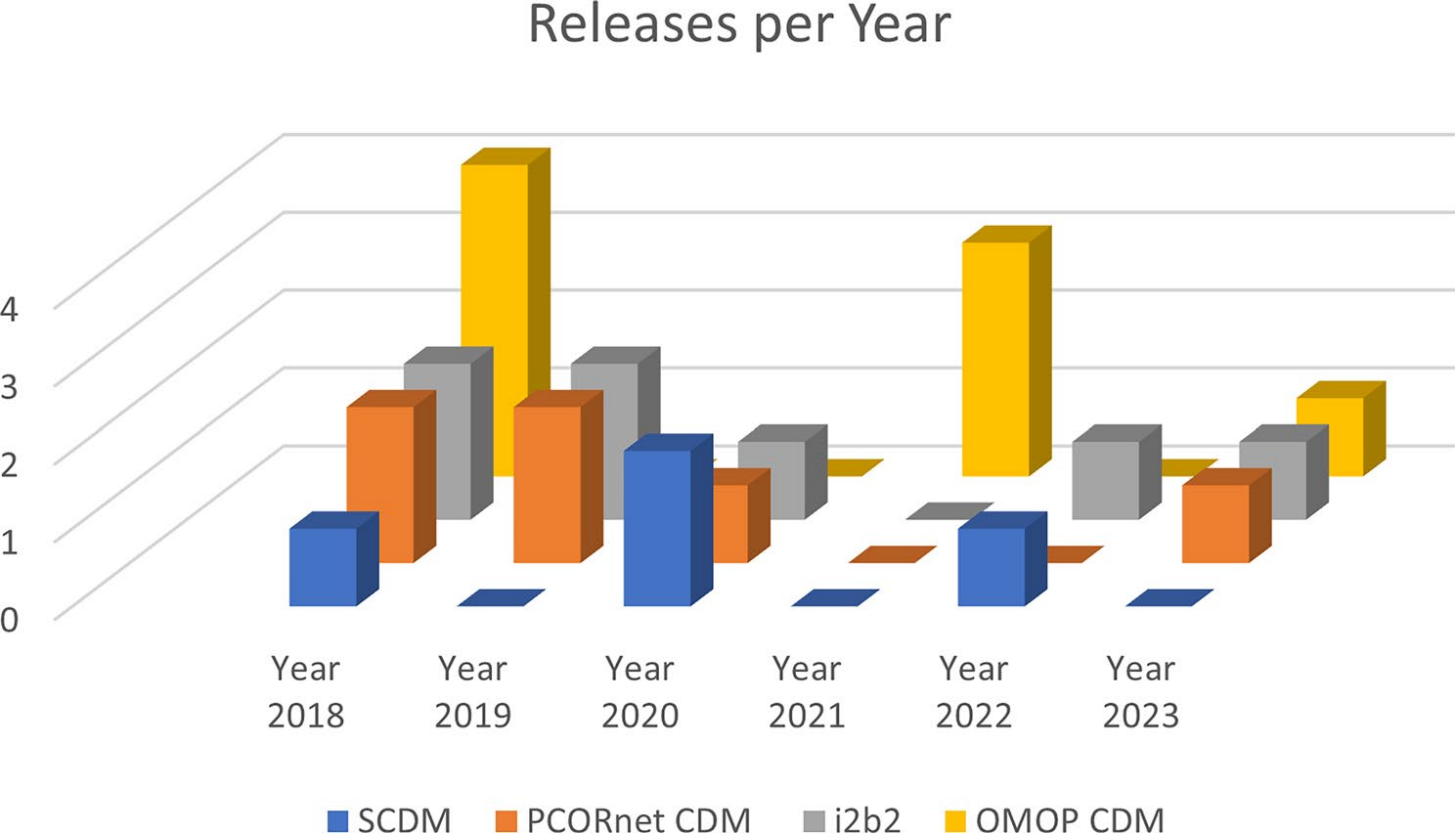
Releases of CDMs in the last 5 years. Regular releases are available [32, 34, 39, 79]

**Fig. 5:**
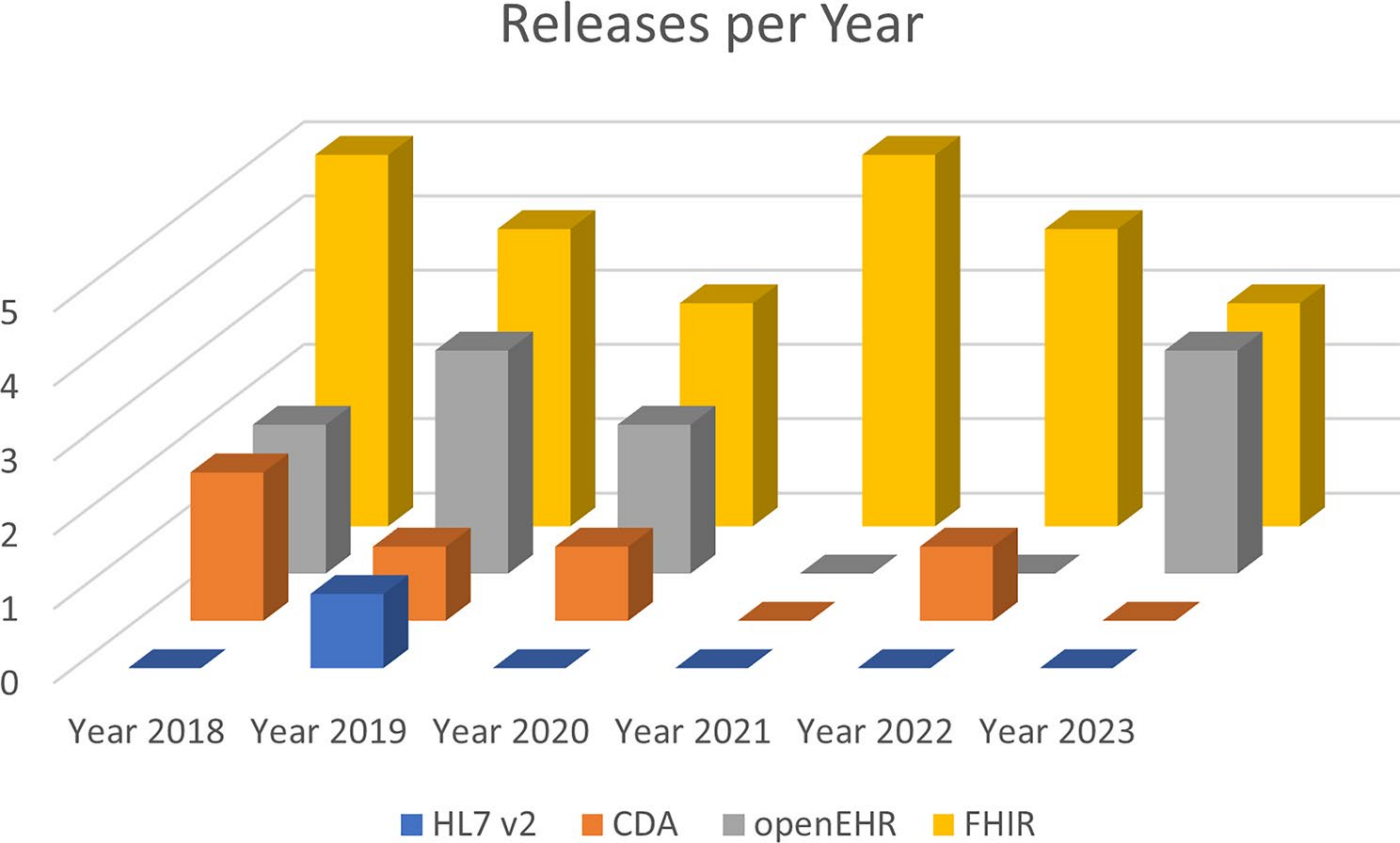
Releases of data standards in the last 5 years. With the exception of HL7 version 2, regular releases are available [44, 55]

In Fig. 4 and Fig. 5 the number of releases in the last five years are visualized. We notice that all CDMs and data standards were updated regularly, rated with $++$, except the HL7 version 2, which was only updated once within this five year, suggesting a $+$ evaluation [32, 39, 44, 55, 79].

### Criterion 4a

The use of standard concepts, although time-consuming and occasionally leading to generalization, is a significant step towards interoperability.

The SCDM primarily preserves original data values. The guiding principles emphasize minimizing data transformation, mapping, manipulation, and combination to ensure that information loss is kept to a minimum [71, 155]. Sentinel utilizes standard terminologies, like SNOMED CT and the ontology framework BioTopLite2 available at all data partners to effectively use the available data [66, 71]. To the best of our knowledge, there is currently no mapping tool available that supports the creation of mappings specifically tailored to SCDM.

PCORnet CDM users are asked to utilize standard vocabulary and terminologies. PCORnet offers several standard terminologies which can be mapped [156], such as SNOMED CT [157], Logical Observation Identifiers Names and Codes (LOINC) [158], rxNorm [159], and International Statistical Classification of Diseases and Related Health Problems (ICD) [160]. A vocabulary mapping software can support the mapping. If a code can be directly mapped to one of the PCORnet CDM standard concept, there is no need to keep the source code [21, 34].

With i2b2, source terminologies are not needed to be mapped to standard terminologies. However, standard concepts, like ARCH (Accessible Research Commons for Health), can be utilized. Yet, i2b2 does not distinguish between standard and local terminologies [39, 143, 161]. SHRINE enables mappings to standard concepts. For instance, Rasmussen et. al [113] performed a real-time mapping of ontology terms to the local i2b2 instance with SHRINE. Within the EuCanImage projects, the OMOP CDM and i2b2 were assessed with a non-image cancer use case [162]. For i2b2 93.6% could be mapped to a standard vocabulary. For the OMOP CDM 92.6% of the codes could be mapped to standard vocabulary.

To ensure institutional interoperability, OMOP CDM users must use the defined OMOP CDM standard vocabulary, for instance SNOMED CT for diagnoses and rxNorm for drugs, but original codes are kept in a provided source field [10]. Many terminologies are integrated in the OMOP CDM as standard concepts, with implemented hierarchies that often facilitate linkages. [10]. The web application ATHENA provides freely available mappings of commonly used vocabularies [42, 163–165]. However, due to Kent and colleagues [10] the greatest challenges is the absence of mapping for local vocabularies. OHDSI encourages the development of custom mappings to the standard concept if necessary [21, 119, 143]. The literature supports the coverage of terminologies used in OMOP CDM for different use cases from various countries, breast cancer in a multi-centric European study [162], cancer registry data in Germany [166]. Within the Criterion 2c we mentioned an ETL process of Yu and colleagues. In [110] they present mapping results on terminologies and concepts from PCORnet CDM to OMOP CDM. Overall, the vocabulary based mapping results were good. The largest vocabulary based loss, with 0.61% was recorded within the drug concept. Garza et al. obtained similar results, limited on their use case, with a terminology coverage of 100 % [15]. To ensure institutional interoperability, OMOP CDM users must use the defined OMOP CDM standard vocabulary, for instance SNOMED CT for diagnoses and rxNorm for drugs, but original codes are kept in a provided source field [10]. Many terminologies are integrated in the OMOP CDM as standard concepts, with implemented hierarchies that often facilitate linkages. [10]. The web application ATHENA provides freely available mappings of commonly used vocabularies [42, 163–165]. However, due to Kent and colleagues [10] the greatest challenges is the absence of mapping for local vocabularies. OHDSI encourages the development of custom mappings to the standard concept if necessary [21, 119, 143]. The literature supports the coverage of terminologies used in OMOP CDM for different use cases from various countries, breast cancer in a multi-centric European study [162], cancer registry data in Germany [166]. Within the Criterion 2c we mentioned an ETL process of Yu and colleagues. In [110] they present mapping results on terminologies and concepts from PCORnet CDM to OMOP CDM. Overall, the vocabulary based mapping results were good. The largest vocabulary based loss, with 0.61% was recorded within the drug concept. Garza et al. obtained similar results, limited on their use case, with a terminology coverage of 100 % [15].

HL7 provides common vocabulary and allow inclusion of external standards and own coding systems for all three standards [45, 167, 168]. Standard coding systems such as SNOMED CT and LOINC enabling compatibility of the CDA with various health and medical fields [132].

OpenEHR allows binding of well-known terminologies, e.g., SNOMED CT, LOINC, provides a set of own mappings, and allows inclusion of external terminologies [138, 142, 169].

Since the SCDM encourages to keep mapping to a minimum and no mapping tool is available, and i2b2 does not distinguish between local and standard concept, we evaluated them with $+$. All the other CDMs and data standards could be rated with $++$, given the terminology policy shown in Table 7.

**Table 7:** CDM terminology availability and recommendations

CDM	Keep Source Voc.	Map to Standard	Mapping Recommendation	Tools
SCDM	$\surd$	$\surd$	Minimize mapping	?
PCORnet		$\surd$	Use standard terms	$\surd$
i2b2	either source or standard		Not required	$\surd$
OMOP	$\surd$	$\surd$	Mandatory mapping	$\surd$
HL7 v2	$\surd$	$\surd$		$\surd$
CDA	$\surd$	$\surd$		$\surd$
FHIR	$\surd$	$\surd$		$\surd$
OpenEHR	$\surd$	$\surd$		$\surd$

### Criterion 4b (Governance of the CDM)

Schneeweiss et al. categorize CDMs into two types: organizing CDMs, which aim to preserve the data in its original form as much as possible, with examples including the SCDM, i2b2, and PCORnet. In contrast, mapping CDMs, such as the OMOP CDM, are designed to transform a given dataset into a standardized set of constructs and analyzable variables. [22]. With strong governance, the OMOP CDM perfectly satisfy Criterion 4b when guidelines are followed [113]. PCORnet CDM is also characterized by strong governance, which emphasizes the importance of not adapting or extending the CDM unless it is absolutely necessary [34]. As organizing CDMs the SCDM and i2b2 CDM are more flexible and less governed.

The HL7 version 2 standard, often referred to as the “non-standard standard,” is widely used but still requires customized adaptations. D’Amore et al. discuss the upsides and barriers of data interoperability with CDA, which offers an improvement over HL7 version 3 with its 3-level definition [45, 170]. Due to the multi-level architecture, data with the based on different templates can still have the same archetype path [171]. Within a use case Lingtong et al. [61] present the semantic interoperability from health care data from different countries. The human and machine readable CDA was employed for standardization and interoperability of the message structure [46, 99]. Existing and reusable CDA templates provide mandatory and optional fields [46, 172].

FHIR addresses challenges faced by CDA and enhances interoperability [53, 173]. FHIR4FAIR, a FHIR implementation guide, supports a FAIR implementation and assessment [100]. Mandatory resources ensure interoperability, but specific requirements can be met by custom resources [45, 174].

The separation of data representation and concept expression in openEHR ensures synaptic interoperability [175]. Archetypes are recognized as the gateway to semantic interoperability [142, 176, 177]. Templates re-use archetypes as building blocks. Additionally, archetype nodes can be limited [138].

With the request to not extend the OMOP CDM or PCORnet CDM, we categorize them with a stronger governance as the i2b2 CDM and SCDM. The HL7 version 2 and CDA lack underlying structure, which is given by openEHR and FHIR, evaluated by $++$.

### Criterion 4c (Data validation)

Criterion 4c is satisfied by Sentinel, which implements a consistent process with over 1,200 data checks and offers a data quality toolkit, including Data Quality Metrics [21, 32, 72, 178]. Every time data is updated, it must undergo extensive quality assurance checks [30, 155].

The PCORnet CDM provides a data curation package with validation and quality checks, employing a two-stage process for data ingestion. Data accessible through PCORnet must conform completeness, including diagnosis codes, plausibility, and persistence. Quality checks include conformance to the PCORnet CDM and its standard terminologies [21, 36].

Wagholikar et al. developed an open-source application for importing electronic health data into the i2b2 platform, which includes data validations among other functionalities [38]. However, a standalone validation tool is to our best knowledge not available.

OHDSI offers various software packages for validation and quality checks, such as Achilles and the Data Quality Dashboard [42]. PEDSnet is a national learning health system and network of children’s hospitals, that offers advanced data quality assessment tools. The OMOP CDM can utilize these tools. Additionally, PEDSnet participate in the data characterization process managed by PCORnet [171, 179, 180].

HL7 version 2 offers a conformance class generator, while validation tools are provided for CDA and FHIR [44, 51, 181].

OpenEHR offers conformance testing to evaluate the quality of solutions and data validation conformance [55]. Additionally, data quality assessment tools like openCQA are introduced by the community [171].

Besides i2b2, all other CDMs and data standards providing a validation or conformance tool are rated with $++$. Wagholikar et al. enable a validation check for i2b2 only within their well maintained ETL pipeline [38] and is therefore evaluated by $+$.

### Criterion 5a

Comprehension can be reached by clear structures, user interfaces, structured and detailed documentation and training. All CDMs for storing and data analysis are table-based and generally easy to understand [15]. Instead of offering a Graphical User Interface (GUI), PopMedNet is integrated into the SCDM. PopMedNet is an open-source informatics platform, designed to support the implementation and operation of distributed health data networks [103]. To explore the SCDM, a synthetic public use file in the SCDM format can be downloaded from the Sentinel website. To be able to run the script, SAS license is required [32, 71]. Sentinel offers documentation within its repository, providing an overview of each table with definitions and data type specifications, along with general guidance [32]. Training materials are freely available on its webpage, and more training can be requested. Sentinel strives to provide the best possible clarity in study planning, execution and reporting [70]. Programs of conducted studies can be accessed through Sentinel [182].

PCORnet offers a centralized access point called “Front Door” that facilitates the submission of research queries to diverse data networks within the PCORnet initiative. Webinars can be accessed without registration [34]. PCORnet CDM guidance is available in the PCORnet forum, which is a GitHub repository of PDF documentations [183]. Additionally, PCORnet states to handle some edge cases, e.g., if the day specific date is not given in the source data [34].

As a patient-centric star schema, the i2b2 is intuitive [114]. All observations, such as diagnoses and medications, are stored in one single table and a public use file is provided [39, 78]. Without comprehensive coding knowledge, i2b2 user can share and query data across institutions with SHRINE and explore data, test hypothesis, and discovery cohorts with the analysis tool tranSMART [143, 184]. i2b2 provides a community wiki that is under development. The i2b2 installation guideline offers detailed information and guidance for some chapters, particularly for developers, while other chapters may be brief or empty. i2b2 encourages participation in working groups and community meetings [39].

OHDSI offers a freely available book, “The Book of OHDSI,” free training through the “EHDEN Academy,” and documentation of the CDM versions [79, 119]. The documentation of some tables, such as the cost table of version 5.4, may be incomplete, and documentation of certain tools may appear outdated, but the majority is detailed and maintained. There are active forums and regular meetings are taking place [42]. Compared to the other CDMs, OMOP CDM contains many tables with linkages between tables. Additionally, OMOP user are encouraged to use the OMOP specific concepts. Consequently, familiarization and transformation is complex [22]. Schneeweiss et. al [22] indicate that analytical tools have faced challenges in generating consistent and valid results.

Data standards have a more intricate structure. HL7 provides specifications and freely available implementation guidance, but the amount of information can be overwhelming. Fee-based training and books are offered by HL7, such as “Principles of Health Interoperability FHIR, HL7 and SNOMED CT” [45]. HL7 version 2 provides predefined XML messages and schemes online [44]. For the CDA, Templates, such as the Continuity of Care Document (CCD) or Consolidated CDA, are provided. Additionally, a freely available implementation guides like ART-DECOR are provided for the CDA [44, 46, 49]. However, it is rather complex and difficult to understand [185]. FHIR is claimed to be more intuitive than CDA and HL7 version 2, with its RESTful API facilitating data input [100, 124, 186]. Public test servers, like HAPI FHIR, a Java API, are provided to support FHIR implementations [60, 181]. A community chat is available for FHIR [44].

openEHR provides clear responsibility alignment through a 3-level approach and offers pre-built data models and pre-integrated platforms for a straightforward and simple architecture [187]. The openEHR Foundation provides a comprehensive information architecture for representing the entirety of EHR content, including pre-built templates [61]. OpenEHR offers the freely available “Guideline Definition Language” and provides a white paper, two short tutorials, and active discussion rooms for support, along with information for development and integration. The provided RESTful API support openEHR users [171, 175, 177]. Low code development of openEHR applications is possible, e.g., a User Interface can be used to form data sets [55, 142]. Even though openEHR offers detailed support and predefined elements, it is known for its complexity. To build a clinical information model, contextual data elements or sensible constraints for archetypes domain and technical experts are needed [171]. Archetypes must be written in the Archetype Definition Language and can be queried by the Archetype Query Language [171, 175].

In summary, SCDM and PCORNet CDM are easy to understand and provide good documentations, whereas i2b2 documentation is incomplete and the OMOP CDM documentation for newer versions sometimes is incomplete and tool documentations are sometimes slightly outdated. HL7 version 2 provides an overwhelming amount of information, CDA and openEHR provide comprehensive documentations and guides but are known for their complexity, therefore we have evaluated them by $+$. As FHIR is more intuitive and supports the implementations, therefore rated by $++$.

### Criterion 5b (Tools)

Sentinel provides routine querying tools, reporting tools, toolkits, software packages, a web-based data visualization, and the Sentinel Query Builder based on SAS [32]. As base of ARIA, Sentinels routine analytic frameworks, known as Sentinel tools, contain reusable SAS-modules [26, 32, 68, 101, 155, 188]. It was shown that the Sentinel tools are capable of reproducing product-outcome association [69, 70]. Some examples tools are: *Medical Product Use Overlap* and *Data Quality Review and Characterization* [26, 101]. Lu et. al [26] developed a publicly available reusable interrupted time series (ITS) tool to assess the impact of regulatory actions on drug use of longitudinal data formatted to the SCDM. Cocoros and colleagues developed a publicly available tool to examine patterns and trends in medication such that the adherence to safe drug use can be assessed [30]. Another tool to analyze manufacturer-level drug utilization was developed by Gagne et. al [188]. Additionally, a data source translation code is available publicly [69].

The PCORnet CDM can make use of tools developed for the Mini SCDM v4.0. In addition, PCORnet provides a curation package, which needs to be passed to become a network partner [36]. Self-developed queries can be distributed through the network by the PopMedNet Query Tool [107]. Waitman et al. [106] introduce a federally compliant, cloud-based data environment for PCORnet and i2b2. i2b2 offers a powerful queering tool [115]. The i2b2 API can be adapted to other CDMs too. For instance, the i2b2 API can be used with PCORnet and OMOP. This offers the great opportunity to use data of these three CDMs together [115, 184]. SHRINE enables population-based research and vocabulary mapping [111, 115].

The i2b2 enable research without comprehensive programming skills and without data leaving the data warehouse [76]. The toolkit Time-based Elixhauser Comorbidity Index (TECI) can be used with i2b2 and OMOP, among others, it can be used for creating mappings between them [151].

OHDSI provides tools to support the ETL process (e.g., USAGI, “White Rabbit”, and “Rabbit in a Hat”) and to apply standardized methods and pipelines for data analysis (e.g., ATLAS and HADES) [10, 42, 189–192].

HL7 offers CDA tools such as C-CDA Online, C-CDA Scorecard, CDA Validator and HL7 C-CDA Online Search Tool [44, 49].

For HL7 version 2 an HL7 application programming interface (HAPI) is provided by HL7 [44].

For FHIR, HL7 offers a range of tools, e.g., for editing, validation, testing, servers, and development, including the Firely editor for constructing FHIR profiles and a tool for mapping of natural language annotations to FHIR [51, 174, 193].

OpenEHR offers open-source and commercial tools for template design and executable medical workflows, e.g., “Better”, a web-based visual design tool [55]. Applications and Software solutions are available for openEHR [171]. For instance HiGHmed introduced a smart infection control system, which connects to an openEHR server and uses standardized, open queries [177].

All CDMs and data standards provide tools to support users and developers. However, to the best of our knowledge, PCORnet supports self-development but lacks complete analysis packages. Additionally, HL7 version 2 is supported solely by HAPI. As a result, we rated these two accordingly by $+$.

### Criterion 5c (Version control)

PCORnet CDM and OMOP CDM version 6 provide limited versioning through history tables [34, 79]. No history tables are available for the SCDM and i2b2 CDM. Sentinel states in their documentation that postal code should be updated by overwriting [32].

HL7 version 2 does not actively support versioning, while CDA provides elements and identifiers for tracking changes [45, 194]. FHIR supports version tracking to prevent conflicts when multiple users edit a resource simultaneously. For large datasets, the “Bulk Data API” is provided as an alternative [51, 195]. When a resource is deleted, a new history entry is created with a “deleted” status, but the resource can be reconstituted as a valid resource. OpenEHR offers change control packages and version control as an integrated part of its architecture [55].

Both FHIR and openEHR present comprehensive solutions, earning them a strong evaluation of $++$. In contrast, PCORnet CDM, OMOP CDM, and CDA provide more limited capabilities, evaluated by $+$. Finally, SCDM, i2b2, and HL7v2 do not support versioning features.

### Limitations

Our comprehensive literature search was done on Scopus, which holds data from a wide range of journals. It is one of the largest and comprehensive academic databases with more than 97.3 million records, 28,300 active serial titles, and 368,000 books [7]. Details regarding the literature search can be found in the Appendix A. Additionally, we restricted our search to the field of medicine and a selected list of search words. Another choice of citation database, categories or keywords might influence the results. Nevertheless, restrictions were kept to a minimum and the choice of threshold is set low to ensure a comprehensive and unbiased literature search.

We assert that we have gathered all pertinent information to the best of our knowledge, utilizing references identified through a comprehensive literature search. However, we acknowledge that the completeness of the literature and information cannot be guaranteed. Nevertheless, we are confident that the most relevant, popular, and frequently utilized references, tools, networks, and related materials have been considered.

Furthermore, we note that a quantitative assessment was often impractical. For example, the number of tools counted does not necessarily reflect their quality and scope. Specifically, generic ETL solutions tend to have a broader impact compared to ETL solutions designed for smaller, less frequently used databases. Therefore, we have decided against quantitative measurements and assessed based on the knowledge gathered from the comprehensive collection of literature.

Infrastructure or network receptively could not be established for all data standards and CDMs. Therefore, the set up was not included in the list of criteria.

## Conclusion

In this article, we discussed various well-established CDMs and data standards, each with its own history, purpose, strengths, and weaknesses. The need for a common data representation has become increasingly apparent in recent years, particularly as diverse health systems across countries require seamless integration.

The COVID-19 pandemic highlighted the critical importance of swift and seamless data exchange and research across borders. For example, FHIR was utilized for the exchange of health information among countries and the Pan American Health Organization, enhancing the surveillance system for adverse events following immunization across the Americas [125]. Chai et al. [196] transformed nine databases from six countries into the OMOP CDM to investigate the reduction of incident cases and the incidence rates of mental health diagnoses during the pandemic.

However, agreeing on a single global common data representation or a national health information exchange network does not seem feasible [185]. Besides the varying scope of application, every CDM and data standard has its strengths and weaknesses, making it better suited to specific use cases or data as highlighted by Schneeweiss et al. [22]. For example, while OMOP CDM accommodates a wide range of data and use cases, its strict adherence to concepts can lead to limited accuracy and information loss during mapping the data with complex relationships [15, 197, 198]. Depending on the specific application and source data, the importance of criteria may vary. If the data has already been standardized into a CDM or a data standard, it is advisable to consider bridging solutions between them rather than opting for costly transformations.

In Table  2 and Table 3 , we indicate whether a CDM or data standard meets various criteria. Some CDMs and data standards particularly excel in certain categories. In its original form, the OMOP CDM accommodates a wide range of data and use cases, while openEHR stands out for its capability to store complex data and support clinical decision-making *(Suitability)*. The popularity of CDMs is largely driven by their accessibility, networking capabilities, and the availability of existing ETL processes. While Sentinel boasts a substantial database, it is primarily limited to the USA. In contrast, OHDSI has collaborators across six continents and maintains a database with over one billion patient records.

Additionally, several ETLs and software solutions for integrating the OMOP CDM are available, contributing to its exceptional *Popularity*. FHIR places a strong emphasis on data exchange, rigorously tested during the COVID-19 pandemic, providing essential building blocks for security measures. In the category of *Adaptability*, the i2b2 CDM excels with its flexible representation, enabling easy modifications to meet specific local needs. All data standards are adaptable, with openEHR and FHIR being particularly extendable through their RESTful APIs.

Interoperability presents a critical challenge that CDMs must tackle. The use of standardized terminologies, such as SNOMED CT and LOINC, ensures compatibility. All models we examined support standard terminologies, and ETL processes enhance interoperability, facilitating seamless data transformation between models. In the category of *Interoperability*, only PCORnet and OMOP meet all three subcriteria within the CDMs. Especially, Garza et al. [15] highlight the strengths of the OMOP CDM within this category. PCORnet provides standard terminologies but does not retain the source code. Conversely, the OMOP CDM enforces strict adherence to its concepts requiring developers to map to these concepts. If local vocabularies cannot be mapped to standard terminologies, due to missing expertise or capacity, this may lead to information loss and failed quality assessments using the data within a distributed network. The data standards FHIR and openEHR also perform equally well in this category. Both data standards enable the inclusion of common standard terminologies and concepts as well as the inclusion of external coding systems. Mandatory FHIR resources enhance interoperability compared to its predecessor, and openEHR eases interoperability by separation of data representation and concept expression. Both offer conformance tools.

Schneeweiss et al. [22] discuss the concept of rule-based transformation and analysis of mapping CDM and its associated complexity. This discussion highlights the need for a comprehensive supporting tool. Therefore, in the final category, *Support*, the OMOP CDM stands out due to its extensive selection of tools, comprehensive documentation, and limited version control. Among all data standards, FHIR is notable for its user-friendliness, which is crucial for broad adoption among healthcare providers.

### Influence of current and future projects

Ongoing and upcoming projects are expected to influence the development and adoption of the CDMs and data standards discussed. In the context of the German and European Union (EU) landscape, we note that the SCDM and PCORNet CDM are not widely used. The tranSMART tool, developed by i2b2, is utilized by the Medical Informatics Initiative [199]. Within the Data Analysis and Real World Interrogation Network (DARWIN) project, the EMA and the European Medicines Regulatory Network have established a coordination center aimed at providing timely and reliable evidence on the use, safety, and effectiveness of medicines, leveraging real-world healthcare databases across the EU [10, 200]. All data within this initiative is standardized to the OMOP CDM. Additionally, EHDEN, a federated network across the EU, adopts the OMOP CDM [120].

Notably, FHIR is prominently featured in several projects and initiatives across Germany and Europe. For instance, the German electronic patient file, the elektronische Patientenakte, is implemented via a FHIR-based Clinical Data Repository. In collaboration with HiGHmed, the Health-X legitimized, open, and federated data platform (dataLOFT) also utilizes FHIR alongside openEHR. HiGHmed highlights the complementary nature of FHIR and openEHR [201, 202]. In contrast, HL7 version 2 and CDA are less common in projects, primarily due to the significant success of FHIR [203].

### Outlook

CDMs and data standards are employed to achieve FAIRness and enhance health research beyond borders. However, health systems vary from country to country CDMs and data standards are influenced by the specific data they are designed for, which might challenge the transformation. For instance, transforming billing information of German claims data into the OMOP CDM might result in information loss [204]. Additionally, frequent changes in national terminologies pose significant challenges. To ensure high CDM adoption, qualitative and comprehensive mappings are essential.

Publicly available ETL processes facilitate simple transformations and encourage collaboration among institutions [22]. However, ETLs also have drawbacks, such as increased effort, additional storage requirements, and the risk of information loss. We have discussed methods to maintain data within its representation while utilizing them together. For example, Shrine enables querying across the i2b2 CDM, OMOP CDM, and PCORnet CDM [161]. OMOPonFHIR allows users to treat an OMOP database like a FHIR server [145]. However, we have encountered some challenges using OMOPonFHIR, including performance issues, incorrect vocabulary mappings, and failures when posing new resources. Addressing these drawbacks can make OMOPOnFHIR a powerful tool for collaborating among institutions that use OMOP CDM for data storage and FHIR for transferring the data.

In the future, tools must be enhanced, and performance improved to advance towards FAIR healthcare. Additionally, the integration of emerging technologies, such as artificial intelligence and machine learning, into CDMs and data standards presents exciting opportunities for more robust data analysis and decision-making. Overall, continued collaboration and innovation will be essential for realizing the full potential of CDMs in healthcare.

## Electronic supplementary material

Below is the link to the electronic supplementary material.

Supplementary material 1

## Data Availability

No datasets were generated or analysed during the current study.

## Abbreviations

ARIA: Active Risk Identification and Analysis
ARCH: Accessible Research Commons for Health
BRIDG: Biomedical Research Integrated Domain Group
CDMH: Common Data Model Harmonization
CDM: Common Data Model
CCD: Continuity of Care Document
CDA: Clinical Document Architecture
dataLOFT: Data legitimized, open and federated
EHR: Electronic Health Records
EMA: European Medicines Agency
ETL: Extracted, Transformed, and Loaded
EU: European Union
FAIR: Findability, Accessibility, Interoperability, and Reusability
FHIR: Fast Healthcare Interoperability Resources
FDA: US Food and Drug Administration
HAPI: HL7 Application Programming Interface
HL7: Health Level Seven
ICD: International Statistical Classification of Diseases and Related Health Problems
IG: Implementation Guide
i2b2: Integrating Biology & the Bedside
ISO: International Organization for Standardization
ITS: Interrupted Time Series
LOINC: Logical Observation Identifiers Names and Codes
OMOP: Observational Medical Outcomes Partnership
OHDSI: Observational Health Data Sciences and Informatics
OAuth: Open Authorization
SCDM: Sentinel CDM
SNOMED: Systematized Nomenclature of Medicine
TECI: Time-based Elixhauser Comorbidity Index
TLS: Transport Layer Security
PCORnet: Patient Centered Outcomes Research Network
